# The Impact of Heat Waves on Health Care Services in Low- or Middle-Income Countries: Protocol for a Systematic Review

**DOI:** 10.2196/44702

**Published:** 2023-10-16

**Authors:** Hadita Sapari, Mohamad Ikhsan Selamat, Mohamad Rodi Isa, Rohaida Ismail, Wan Rozita Wan Mahiyuddin

**Affiliations:** 1 Department of Public Health Medicine Faculty of Medicine Universiti Teknologi Mara Selangor Malaysia; 2 Environmental Health Research Centre, Institute for Medical Research National Institutes of Health Ministry of Health Selangor Malaysia

**Keywords:** heat wave, burden, health care service, morbidity, low- or middle-income countries, LMICs, mortality

## Abstract

**Background:**

Heat waves significantly impact ecosystems and human health, especially that of vulnerable populations, and are associated with increased morbidity and mortality. Besides being directly related to climate-sensitive health outcomes, heat waves have indirectly increased the burden on our health care systems. Although the existing literature examines the impact of heat waves and morbidity, past research has mostly been conducted in high-income countries (HICs), and studies on the impact of heat waves on morbidity in low- or middle-income countries (LMICs) are still scarce.

**Objective:**

This paper presents the protocol for a systematic review that aims to provide evidence of the impact of heat waves on health care services in LMICs.

**Methods:**

We will identify peer-reviewed studies from 3 online databases, including the Web of Science, PubMed, and SCOPUS, published from January 2002 to April 2023, using the PRISMA (Preferred Reporting Items for Systematic reviews and Meta-Analyses) guidelines. Quality assessment will be conducted using the Navigation Guide checklist. Key search terms include *heatwaves*, *extreme heat*, *hospitalization*, *outpatient visit*, *burden*, *health services*, and *morbidity*.

**Results:**

This systematic review will provide insight into the impact of heat waves on health care services in LMICs, especially on emergency department visits, ambulance call-outs, hospital admissions, outpatient department visits, in-hospital mortality, and health care operational costs.

**Conclusions:**

The results of this review are anticipated to help policymakers and key stakeholders obtain a better understanding of the impact of heat waves on health care services and prioritize investments to mitigate the effects of heat waves in LMICs. This entails creating a comprehensive heat wave plan and ensuring that adequate infrastructure, capacity, and human resources are allocated in the health care sector. These measures will undoubtedly contribute to the development of resilience in health care systems and hence protect the health and well-being of individuals and communities.

**Trial Registration:**

PROSPERO CRD42022365471; https://www.crd.york.ac.uk/prospero/display_record.php?RecordID=365471

**International Registered Report Identifier (IRRID):**

DERR1-10.2196/44702

## Introduction

The world is facing significant climate change with a variety of impacts, especially from rising temperatures and more frequent heat wave episodes. Globally, the annual average temperature has been rising since the beginning of the 20th century, and it is now higher than the 1901 to 2000 average [1]. Global warming as a result of the continuous increase in greenhouse gas production has contributed to heat waves with increasing frequency, severity, and duration [2]. Moreover, the effects of heat waves are aggravated by rapid urbanization, population growth, and the urban heat island effect [3]; more than half of the world’s population lived in urban settings in 2017 [4]. The Intergovernmental Panel on Climate Change (IPCC) has projected that global warming of 1.5 °C could be reached in the 2030s if countries do not adhere to the Paris Agreement and do not create a mitigation action plan [5,6]. Major heat wave events have caused widespread casualties and strain on health care systems worldwide. The impacts were observed during the 2003 European heat wave, with the deaths of an estimated 72,000 people, and the 2010 Russian heat wave, with 56,000 deaths [7]. The number of deaths is projected to increase in the future, with 250,000 additional deaths per year between 2030 and 2050 [8]. An extended time-series study demonstrated increased risks of mortality of 5.1%, 2.9%, and 5.5% for every degree rise in temperature above the identified threshold for London, Budapest, and Milan, respectively [9].

Heat waves are generally defined as prolonged periods of exceptionally high or extreme temperatures and are characterized by the magnitude, duration, severity, and extent of the event [10,11]. Up to now, there has been no agreed definition of a heat wave, as the best definition would largely depend on the specific climatic zone of a geographical area, time trends, and individual acclimatization [12]. The unavailability of a unified global index further complicates research that seeks to compare results [12] and discuss patterns, trends, and impacts [13] for the development of health warning systems. The World Meteorological Organization and the Centre for Research on the Epidemiology of Disasters define heat waves as 2 or more consecutive days of temperatures exceeding the local region’s given temperature or a percentile threshold [14-16]. A heat wave index (HI) can be defined using temperatures alone or in combination with other meteorological factors, such as humidity and wind speed, for a specified duration, ranging from 1 to several consecutive days [10,13]. Some researchers prefer to use daily mean temperature [17], while others have used daily maximum and apparent temperature [18] to understand the exposure relationship. For instance, Kent et al [12] and Zhang et al [19] found a relative mean temperature HI was the most predictive of adverse heat-related health effects compared to other HIs. According to Wu et al [20], the diversity in heat wave definitions could lead to uncertainty of approximately 22% in predictions of mortality risk.

An increase of 1 °C in temperature above a city-specific threshold is a serious public health concern due to increased mortality and hospital admissions [21-23]. Health effects following heat waves are usually acute, ranging from immediate effects to effects that persist until 7 days later [24,25]. Heat waves have been linked directly to multiple medical conditions, such as heat-related illnesses [26], accelerated complications and deaths from cardiorespiratory and renal diseases, endocrine disorders, food and waterborne diseases, vector-borne diseases [27], accidents [28], increased preterm births [12], and many more. Furthermore, morbidity and mortality in urban areas are higher due to the urban heat island effect [29,30]. On the other hand, improvements in living conditions, urban green space, and awareness of heat waves [31] have been shown to reduce the adverse health effects.

Heat waves will create a surge in demand for health care services and subsequently increase the burden on these services at all levels, including primary care, elective admissions, and intensive care. This includes increased ambulance call-outs, slower response time [32], increased emergency and outpatient department visits [33], increased hospitalization [34], shortages of medicine, and ultimately increased health care expenditures, both direct and indirect [35]. Even though a few previous studies found that emergency hospital admissions were lower compared to mortalities during heat waves [36,37], the number of excess emergency department visits can still be high and thus impose an extra burden on health care services. For instance, during heat wave events in California in 2006, an excess of 16,166 emergency department visits was documented [38]. Health care systems will be under pressure with high bed occupancy rates and constrained resources, which may ultimately affect the care plans of patients [39] and lead to greater economic costs [40].

The effects of heat waves are more pronounced in vulnerable groups. Numerous systematic reviews have examined the effects of heat waves on older people [31]; children [41,42]; men and women [43]; pregnant women [44]; people with chronic conditions, such as mental illness [45,46], cardiovascular disease [47-50], and kidney disease [51]; people experiencing homelessness; people with low socioeconomic status and poor access to green areas [52]; and people in certain occupational sectors, such as agriculture, forestry or fishing, construction, and manufacturing [53]. Reviews that examined the impact of heat waves on health services in low- or middle-income countries (LMICs) are still scare [52,54], with the majority concentrating on high-income countries (HICs) [34] or a combination of LMICs and HICs [55,56]. Moreover, LMICs have limited access to health care systems, lack of adequate infrastructure, and a lower allocation of gross domestic product (GDP) to health care [52]; they also have more vulnerable populations due to low adaptive capacity to heat waves [57-59], lower socioeconomic status, and lack of cooling facilities such as air-conditioning in homes [60,61].

Thus, it is necessary to understand the specific, isolated impact of heat waves on health care services in LMICs in order to ensure adequate preparedness, health promotion, and risk management and allow preventive measures to be carried out effectively to protect vulnerable people from the harmful effects of extreme weather, as highlighted by the World Health Organization and the US National Climate Assessment Program 2014 [62]. Moreover, previous research in China has demonstrated that public health divisions and clinical divisions were unprepared to act confidently in facing challenges related to climate change due to lack of logistical support, capacity building, and funding [27,63]. The enhancement of health care services ultimately aims to reduce morbidity and mortality arising from heat wave exposure.

This systematic review aims to evaluate the impact of heat waves on health care services in LMICs. Moreover, this study aims to identify critical research gaps in the existing literature for future research and to aid in the dissemination of important research findings to policymakers and relevant stakeholders for strategic policy planning and implementation.

## Methods

### Protocol Registration

This study has been registered with the international Prospective Register of Systematic Reviews (PROSPERO; CRD42022365471). This paper presents a protocol for a systematic review of literature reporting on the impact of heat waves on health care services, particularly in LMICs.

### Identifying the Research Question

The main research question is “How do heat waves place a burden on health care systems, particularly in LMICs?” The specific questions are (1) “What are the impacts of heat waves on health care systems—emergency department visits, hospital admissions, outpatient department visits, ambulance call-outs, in-hospital mortality, and health care operational costs?” and (2) “What are the risk factors involved?”

### Search Strategy

**Figure 1:**
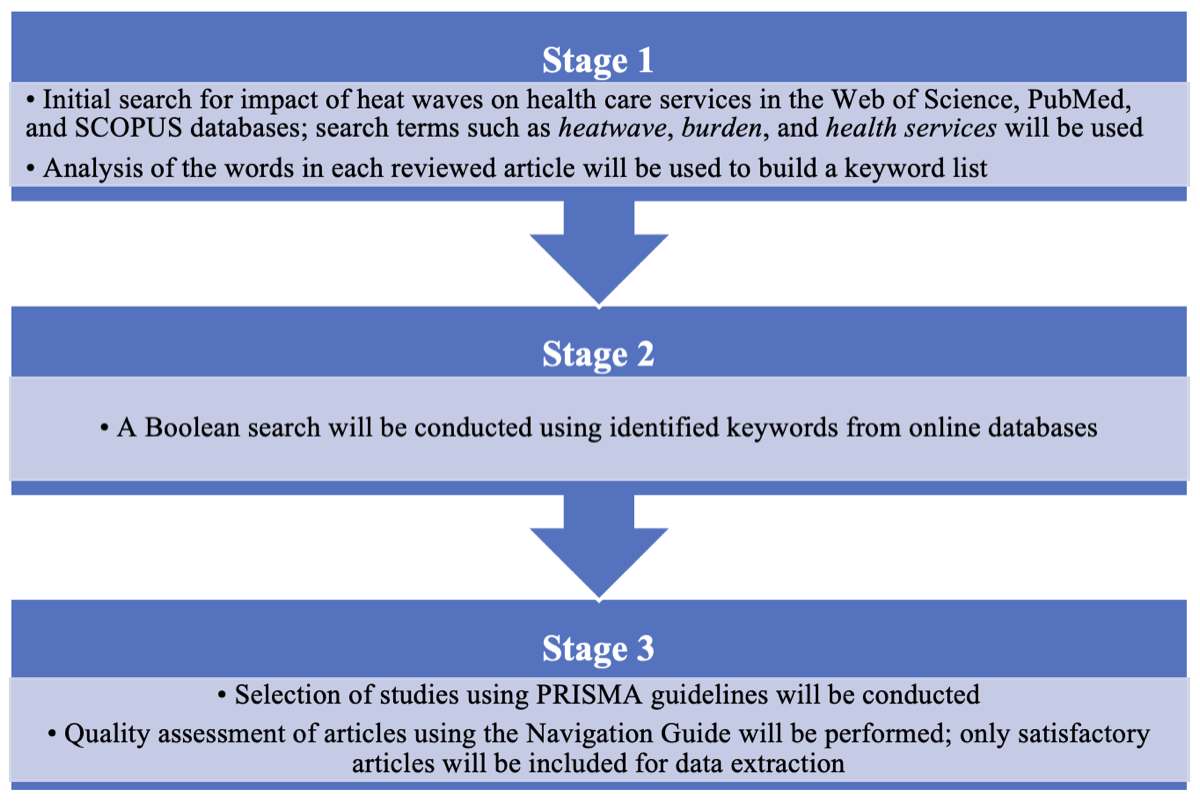
Schematic flow chart of the systematic review process. PRISMA: Preferred Reporting Items for Systematic reviews and Meta-Analyses.

A comprehensive literature search of electronic databases for peer-reviewed literature published between January 2002 and April 2023 will be conducted on the Web of Science, PubMed, and SCOPUS using a common search strategy. The process of the review started in March 2023 and is currently still in the screening phase. This process involves a 3-stage protocol, as depicted in Figure 1, using the Boolean operators “AND” and “OR.” A search strategy combining MeSH terms and keywords that will be used in the 3 online databases is described in Multimedia Appendix 1. As a preliminary search, these keywords will be used on each database using the following search terms: (“heat wave” OR “extreme heat” OR “heat wave”) AND (“hospitalization” OR “hospital admission”) OR (“morbidity” OR “heat-related illness”) OR “ambulance” OR “outpatient visit” OR “emergency department” OR “health services.”

### Selection of Eligible Studies

To increase the feasibility of this review, studies that have been published during the past 20 years will be included. Subsequently, eligibility criteria will be developed using the PECO (population, exposure, comparison, and outcomes) analytical framework, as detailed in Textbox 1.

**Inclusion and exclusion criteria, developed using the PECO (population, exposure, comparison, and outcomes) analytical framework.**

**Inclusion criteria**

- Population: The general population of low- or middle-income countries (LMICs) that are affected by heat waves and seek treatment in health care services, regardless of their age, gender, location, or ethnicity. The International Monetary Fund definition of LMICs will be used [64,65]; the list of included countries is provided in Multimedia Appendix 1.
- Exposure: All studies related to heat waves will be included, regardless of their definition of a heat wave.
- Comparison: The same population at a time they are not exposed to a heat wave event or a comparable population that are not exposed to a heat wave event.
- Outcomes: Health care services, including ambulance-call-outs, emergency department visits, outpatient department visits, hospitalization, and in-hospital mortality.

**Exclusion criteria**

- Population: Morbidity and mortality outside of health care services (ie, hospitals, outpatient clinics, and ambulance dispatches); populations in high-income countries
- Exposure: No definition of heat wave provided; studies on ambient temperature that do not examine heat waves; studies on cold temperatures

All observational studies on heat waves will be included. Intervention studies (eg, randomized controlled trials, single-arm studies), case reports, guidelines, book chapters, commentaries, reviews (eg, systematic reviews, scoping reviews), editorials, protocols, short communications, guidelines, surveillance studies, qualitative research, and non-English publications will be excluded. The full text of selected studies is required to be retrievable for further review. The epidemiological measures must be reported as relative risks, odds ratios, or incidence rate ratios with the 95% CI.

### Study Selection Process

Before starting the screening process, a standardized questionnaire for study selection will be developed based on the predetermined eligibility criteria followed by a training session at each level of screening to ensure high interrater reliability (>75% agreement).

Eligible articles will be exported into EndNote X9 (Clarivate) for data management, which will include identifying and removing duplicates, by one reviewer. Subsequently, the data will be exported into Covidence (Level 10) for screening and data extraction. All attempts will be made to obtain the full text of selected articles, including web searching, engaging with the University Teknologi Mara librarian, or, if necessary, contacting the author.

After removing all duplicate articles, 2 reviewers will examine the eligibility of the articles in a 2-stage process. During the first screening, the list of titles and abstracts in the search results will be evaluated for relevancy. In the second screening, potentially relevant full-text papers will be reviewed according to the eligibility criteria. Articles that do not fulfill the criteria will be excluded from the study. Three reviewers will independently execute both stages of study selection, and cross-validation will be conducted for any potential disagreements. Cross-validation will be used to analyze inconsistencies in the list of included and excluded studies. In the event of a disagreement, a fourth reviewer will be assigned before considering the involvement of a fifth verifier. All disagreements will be resolved through discussion, and agreement will be assessed using the κ statistic, with a value of more than 0.8 considered good agreement.

The selection process will be conducted following the PRISMA-P (Preferred Reporting Items for Systematic reviews and Meta-Analyses protocol) checklist [66]. We expect that this systematic review will be completed in December 2023.

### Data Items and Data Abstraction Process

Three independent reviewers will perform data abstraction to ensure high interrater reliability and minimize error. Prior to this process, a standardized data collection form will be pilot-tested by 3 independent reviewers. Any discrepancies identified will be resolved by a fourth and fifth reviewer.

Data items will include all relevant information on (1) study characteristics (author, publication year, study design, study location, study years); (2) exposure definition; (3) population characteristics (study population geographical area, vulnerability factors such as age, gender, medical conditions), (4) measures of exposure and outcome, (5) confounders, and (6) effect estimates and outcome characteristics (ambulance call-outs, emergency department visits, outpatient department visits, and in-hospital admissions).

### Charting the Data

A data charting form will be used to capture all relevant information for each included study in the review. This process will be conducted by 2 independent reviewers. A thematic analysis approach will be used to identify the key concepts from the full-text articles and code them into non–mutually exclusive themes. All relevant articles will be coded manually in the spreadsheets and presented in the evidence table. The third reviewer will verify the charted data.

### Critical Appraisal

#### Overview

The included papers will be assessed in accordance with the Navigation Guide, which provides criteria for evaluating the quality of systematic reviews of environmental health observational studies in order to minimize the risk of bias (RoB) and maximize transparency [67]. The assessment will be conducted in three stages: (1) individual study RoB assessments, (2) assessment of the overall quality of the evidence across all included papers, and (3) rating of the overall strength of the evidence across studies. This process will be independently conducted by 2 reviewers and followed by discussion to reach agreement on ratings. Whenever there is disagreement, a third reviewer will be called in for further discussion on the appraisal.

#### RoB Assessment

The quality of the individual studies will be assessed using the Office of Health Assessment and Translation (OHAT) RoB assessment tool to examine internal validity and whether the study’s design and execution jeopardized the validity of the association between exposure and outcome [68]. This tool was adapted from several guidelines, such as those of the Agency of Health Care Research and Quality [69], the Navigation Guide [70] and the Cochrane Handbook [71]. Since OHAT does not specifically consider time-series environmental health study design, 2 subject-matter experts will modify some of its domains according to our research questions. The RoB domains that will be used are selection, confounding, exposure assessment, outcome assessment, selective reporting, conflict of interest, and other possible bias. The overall rating will be categorized as “definitely low,” “probably low,” “probably high,” or “definitely high.”

#### Assessment of Overall Quality of Evidence Across All Included Papers

Based on the Navigation Guide, the certainty of evidence will be assessed using the Grading of Recommendation Assessment, Development and Evaluation (GRADE) system for each exposure outcome. The body of evidence will be initially graded as moderate based on priority criteria in human observational evidence [67] and then downgraded according to 5 domains (individual study limitations, inconsistency of results, indirectness of evidence, imprecision, and publication bias) and upgraded based on 3 domains (dose-response gradient, large magnitude of effect, and confounding). The final assessment will be as “high,” “moderate,” “low,” or “very low” [72].

#### Rating of Overall Strength of Evidence Across Studies

The Navigation Guide further outlines four considerations when rating the overall strength of evidence across studies [67], which include (1) the quality of the body of evidence, (2) the direction of effect estimates, (3) confidence in the effect estimates, and (4) any other attributes that might affect certainty. The rating will be either “sufficient,” “limited,” “inadequate,” or “evidence of lack of effect.” The final rating will indicate the level of certainty of effect [70].

### Ethical Approval

Formal ethical approval is not required as this study does not use primary data.

## Results

We anticipate publishing the systematic review by May 2024. To enhance the transparency and clarity of the results and provide a comprehensive overview of the results obtained from each database during preliminary screening, we present our current results in Table 1, which includes a few examples.

**Table 1:** Characteristics of the studies during preliminary screening.

Title	Year	Journal name	Database	First screening	Second screening	Reason for exclusion
“Cause-specific risk of hospital admission related to extreme heat in older adults” [73]	2014	*JAMA*	SCOPUS	Included	Excluded	Conducted in high-income country
“Increased risk of emergency hospital admissions for children with renal diseases during heatwaves in Brisbane, Australia” [74]	2014	*World Journal of Pediatrics*	SCOPUS	Excluded	N/A^a^	Conducted in high-income country
“The effects of heatwaves on human morbidity in primary care settings: a case-crossover study” [75]	2022	*Scholarly Community Encyclopedia*	Web of Science	Excluded	N/A	Short communication
“The burden of extreme heat and heatwave on emergency ambulance dispatches: A time-series study in Huainan, China” [76]	2016	*Science of the Total Environment*	PubMed	Included	Included	N/A
“Burden of non-accidental mortality attributable to ambient temperatures: a time series study in a high plateau area of southwest China” [77]	2019	*BMJ Open*	PubMed	Included	Excluded	Mortality outside health care services

^a^N/A: not applicable.

### Descriptive Analysis

**Figure 2:**
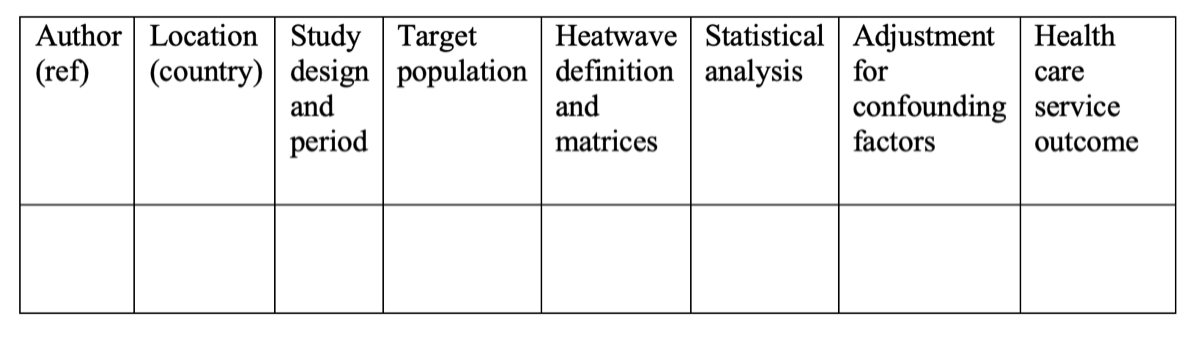
Sample table for characteristics of articles selected for the study.

**Figure 3:**
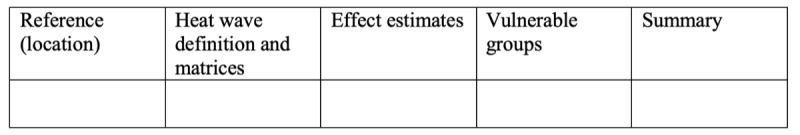
Sample table for impact on hospital admissions.

**Figure 4:**
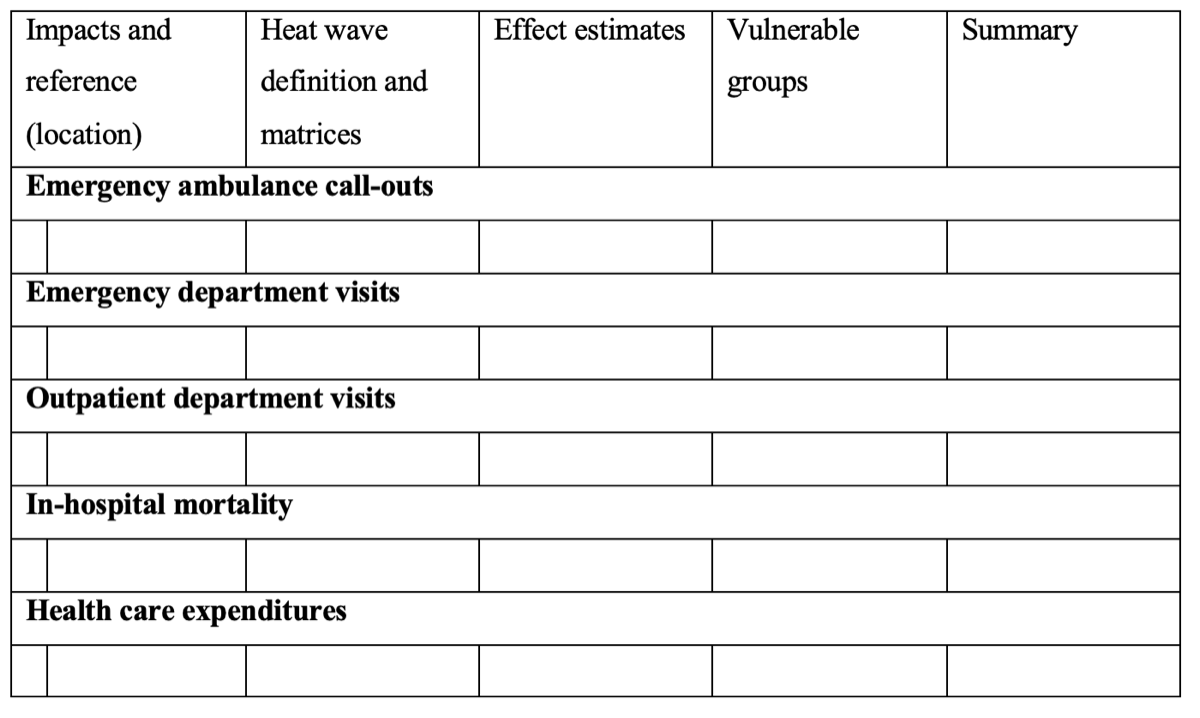
Sample table for impact of heat waves on other health care services.

A narrative report will be produced to summarize the extracted data related to the research questions in the final review. The results will be presented in the final evidence tables; Figures 2, 3, and 4 represent samples of these tables, which include the following information: (1) the study characteristics, including author, publication year, study design, study location, the years the study covers, and the definition of a heat wave; (2) population characteristics and vulnerability (ie, risk factors), including age, gender, race, location (urban vs rural); (3) type of disease according to body system, such as cardiovascular disease, respiratory disease, and renal disease, which will be presented in the effect estimates sections of the tables in Figures 3 and 4; and (4) the burden on health care services, including ambulance call-outs, emergency department visits, outpatient department visits, hospital admissions, in-hospital mortality, and health care operational costs.

### Statistical Analysis

This study will not conduct a meta-analysis of the results due to the heterogeneity of outcome measures. Hence, only descriptive thematic analysis will be performed. Nevertheless, the RoB assessment will be performed using SPSS (version 27; IBM Corp) for categorical outcomes, which will be presented as frequencies and percentages.

## Discussion

### Principal Findings

It is anticipated that the strain on health care systems will increase significantly if heat wave events rise in frequency, length, and severity. This review’s findings are expected to demonstrate that heat waves significantly increase ambulance call-outs, emergency department visits, hospitalization, outpatient department visits, and in-hospital mortality, as well as health care operational costs. Previously, Campbell et al [78] found that ambulance call-outs increased by 34% during extreme heat waves, 10% during severe heat waves, and 4% during low-intensity heat waves. Davis and Novicoff [79] found significantly increased risk for emergency department visits and hospitalization for White and Black people, as well as men and women aged 20 to 49 years, with a variety of presentations, including diabetes, pregnancy complications, poisoning, and traffic crashes, [80] in addition to common presentations such as renal disorder [81,82], cardiovascular disease, respiratory disease [83,84], mental disease [85], and heat-related illness [86].

To our knowledge, this study will be the first to review the impacts of heat waves on health care systems in LMICs and thus fill a knowledge gap. Since the impact of heat waves is more pronounced in LMICs due to their inadequate infrastructure, low access to health care [52], and dense populations of vulnerable people [87], it will be beneficial if LMICs can identify temperature thresholds and vulnerable groups in their region so that targeted interventions can be implemented effectively. Moreover, morbidity indicators such as ambulance call-outs, hospital admissions, and emergency department visits should be used during heat wave impact assessments and the development of early warning systems. Wang et al [88] recommended examining ambulance dispatch data to develop a real-time surveillance heat health warning system that would allow early detection of residents’ health demands and health status in a region. Toloo et al [89] demonstrated that the implementation of an early warning system can be cost-effective and reduce mortality and ambulance call-outs. Indeed, many HICs have developed and implemented heat health action plans [90,91]. However, Ahmedabad, India, has become the first South Asian city to develop and implement an early warning system and heat preparedness plan based on collected evidence, such as an analysis of local temperature and mortality data and surveys of the population vulnerable to heat. Nevertheless, evaluation and continuous improvement are still ongoing [92].

The findings of this review also could assist public health and health care systems in allocating resources effectively, such as by allocating more GDP to health care, improving existing infrastructure [26], increasing human resources, and promoting health. Apart from this, health care workers must be provided with adequate training and updates on the latest information or guidelines related to heat waves [16]. Enhanced diagnostic accuracy and speed, followed by immediate treatment, is vital for hospitals and other community medical institutions in order to reduce morbidity and mortality [26]. Lastly, understanding the impact of heat waves on health care services underscores the need for climate change mitigation and adaptation, such as increasing the resilience of health care systems and being responsive to the effects of heat waves [93,94].

Among the limitations that we expect in this review are the inability to access full texts and some of the articles not being in English. This could limit the overall findings of the review. Secondly, during our search, we found numerous articles that examined the impact of ambient temperature on health care services but few that examined the impact of heat waves. In fact, some of the articles did not provide a definition of a heat wave. This might be due to underrepresentation of some LMICs [95], which will further limit the findings of the review. However, we believe that this finding could encourage these countries to participate and conduct more research on this topic, as well as highlight to HICs that funding to these countries is necessary for them to conduct more research.

### Conclusion

Undeniably, heat waves pose a significant health risk to individuals and the community. Therefore, understanding the burden on health care services due to exposure to heat waves is of paramount importance as heat waves are becoming more frequent and increasing in intensity. This proposed systematic review is expected to provide a better understanding of the impact of heat waves on health care systems in LMICs; this will be critical for optimal planning, preventive activities, policymaking, and climate action. By prioritizing this understanding and disseminating this information to policymakers and relevant stakeholders, we can better protect individuals’ and communities’ health and well-being, as well as construct a more resilient health care system in the face of climate change.

## Appendix

Multimedia Appendix 1 Search strategy, list of low- and middle-income countries, and risk of bias assessment.

Multimedia Appendix 2 PRISMA-P (Preferred Reporting Items for Systematic reviews and Meta-Analyses protocol) checklist.

## Abbreviations

GDP: gross domestic product
GRADE: Grading of Recommendation Assessment, Development and Evaluation
HI: heat wave index
HIC: high-income country
IPCC: Intergovernmental Panel on Climate Change
LMIC: low- or middle-income country
OHAT: Office of Health Assessment and Translation
PECO: population, exposure, comparison, and outcomes
PRISMA-P: Preferred Reporting Items for Systematic reviews and Meta-Analyses–Protocols
PROSPERO: Prospective Register of Systematic Reviews
RoB: risk of bias
