# Supplementary material for: The Impact of Heat Waves on Health Care Services in Low- or Middle-Income Countries: Protocol for a Systematic Review
Source: JMIR Res Protoc. 2023 Oct 16;12:e44702. doi: 10.2196/44702 (PMC10616749; doi:10.2196/44702)
Supplement: Multimedia Appendix 1 [file resprot_v12i1e44702_app1.docx]

**S2. Preliminary pilot search strategy in WEB OF SCIENCE (WOS) , PUBMED and SCOPUS**

| **Databases** | **Search items**  **#1** (("heatwave"[All Fields] OR "heatwaves"[All Fields]) AND ("hospital s"[All Fields] OR "hospitalisation"[All Fields] OR "hospitalization"[MeSH Terms] OR "hospitalization"[All Fields] OR "hospitalising"[All Fields] OR "hospitality"[All Fields] OR "hospitalisations"[All Fields] OR "hospitalised"[All Fields] OR "hospitalizations"[All Fields] OR "hospitalized"[All Fields] OR "hospitalize"[All Fields] OR "hospitalizing"[All Fields] OR "hospitals"[MeSH Terms] OR "hospitals"[All Fields] OR "hospital"[All Fields]) AND ("burden"[All Fields] OR "burdened"[All Fields] OR "burdening"[All Fields] OR "burdens"[All Fields]) AND ("health services"[MeSH Terms] OR ("health"[All Fields] AND "services"[All Fields]) OR "health services"[All Fields] OR ("health"[All Fields] AND "service"[All Fields]) OR "health service"[All Fields])) AND (2002:2022[pdat])  **#2** (("heatwave"[All Fields] OR "heatwaves"[All Fields]) AND ("ambulance s"[All Fields] OR "ambulances"[MeSH Terms] OR "ambulances"[All Fields] OR "ambulance"[All Fields]) AND ("burden"[All Fields] OR "burdened"[All Fields] OR "burdening"[All Fields] OR "burdens"[All Fields]) AND ("health services"[MeSH Terms] OR ("health"[All Fields] AND "services"[All Fields]) OR "health services"[All Fields] OR ("health"[All Fields] AND "service"[All Fields]) OR "health service"[All Fields])) AND (2002:2022[pdat])  #3 (("heatwave"[All Fields] OR "heatwaves"[All Fields]) AND ("economics"[MeSH Subheading] OR "economics"[All Fields] OR "cost"[All Fields] OR "costs and cost analysis"[MeSH Terms] OR ("costs"[All Fields] AND "cost"[All Fields] AND "analysis"[All Fields]) OR "costs and cost analysis"[All Fields]) AND ("burden"[All Fields] OR "burdened"[All Fields] OR "burdening"[All Fields] OR "burdens"[All Fields]) AND ("health services"[MeSH Terms] OR ("health"[All Fields] AND "services"[All Fields]) OR "health services"[All Fields] OR ("health"[All Fields] AND "service"[All Fields]) OR "health service"[All Fields])) AND (2002:2022[pdat])  #4 (("heatwave"[All Fields] OR "heatwaves"[All Fields]) AND ("hospital s"[All Fields] OR "hospitalisation"[All Fields] OR "hospitalization"[MeSH Terms] OR "hospitalization"[All Fields] OR "hospitalising"[All Fields] OR "hospitality"[All Fields] OR "hospitalisations"[All Fields] OR "hospitalised"[All Fields] OR "hospitalizations"[All Fields] OR "hospitalized"[All Fields] OR "hospitalize"[All Fields] OR "hospitalizing"[All Fields] OR "hospitals"[MeSH Terms] OR "hospitals"[All Fields] OR "hospital"[All Fields]) AND ("admission"[All Fields] OR "admissions"[All Fields]) AND ("burden"[All Fields] OR "burdened"[All Fields] OR "burdening"[All Fields] OR "burdens"[All Fields]) AND ("health services"[MeSH Terms] OR ("health"[All Fields] AND "services"[All Fields]) OR "health services"[All Fields] OR ("health"[All Fields] AND "service"[All Fields]) OR "health service"[All Fields])) AND (2002:2022[pdat])  **#5** (("heatwave"[All Fields] OR "heatwaves"[All Fields]) AND ("outpatient s"[All Fields] OR "outpatients"[MeSH Terms] OR "outpatients"[All Fields] OR "outpatient"[All Fields]) AND ("visit"[All Fields] OR "visitation"[All Fields] OR "visitations"[All Fields] OR "visited"[All Fields] OR "visiting"[All Fields] OR "visits"[All Fields])) AND (2002:2022[pdat])  **#6** (("extreme heat"[MeSH Terms] OR ("extreme"[All Fields] AND "heat"[All Fields]) OR "extreme heat"[All Fields]) AND ("epidemiology"[MeSH Subheading] OR "epidemiology"[All Fields] OR "morbidity"[All Fields] OR "morbidity"[MeSH Terms] OR "morbid"[All Fields] OR "morbidities"[All Fields] OR "morbids"[All Fields]) AND ("hot temperature"[MeSH Terms] OR ("hot"[All Fields] AND "temperature"[All Fields]) OR "hot temperature"[All Fields] OR "heat"[All Fields]) AND ("family"[MeSH Terms] OR "family"[All Fields] OR "relation"[All Fields] OR "relatability"[All Fields] OR "relatable"[All Fields] OR "related"[All Fields] OR "relates"[All Fields] OR "relating"[All Fields] OR "relational"[All Fields] OR "relations"[All Fields]) AND ("cost of illness"[MeSH Terms] OR ("cost"[All Fields] AND "illness"[All Fields]) OR "cost of illness"[All Fields] OR ("illness"[All Fields] AND "burden"[All Fields]) OR "illness burden"[All Fields]) AND ("health services"[MeSH Terms] OR ("health"[All Fields] AND "services"[All Fields]) OR "health services"[All Fields] OR ("health"[All Fields] AND "service"[All Fields]) OR "health service"[All Fields])) AND (2002:2022[pdat])  **#7** (("extreme heat"[MeSH Terms] OR ("extreme"[All Fields] AND "heat"[All Fields]) OR "extreme heat"[All Fields]) AND ("epidemiology"[MeSH Subheading] OR "epidemiology"[All Fields] OR "morbidity"[All Fields] OR "morbidity"[MeSH Terms] OR "morbid"[All Fields] OR "morbidities"[All Fields] OR "morbids"[All Fields]) AND ("hot temperature"[MeSH Terms] OR ("hot"[All Fields] AND "temperature"[All Fields]) OR "hot temperature"[All Fields] OR "heat"[All Fields]) AND ("family"[MeSH Terms] OR "family"[All Fields] OR "relation"[All Fields] OR "relatability"[All Fields] OR "relatable"[All Fields] OR "related"[All Fields] OR "relates"[All Fields] OR "relating"[All Fields] OR "relational"[All Fields] OR "relations"[All Fields]) AND ("illness"[All Fields] OR "illness s"[All Fields] OR "illnesses"[All Fields]) AND ("emergency service, hospital"[MeSH Terms] OR ("emergency"[All Fields] AND "service"[All Fields] AND "hospital"[All Fields]) OR "hospital emergency service"[All Fields] OR ("emergency"[All Fields] AND "department"[All Fields]) OR "emergency department"[All Fields])) AND (2002:2022[pdat])  **#8** (("extreme heat"[MeSH Terms] OR ("extreme"[All Fields] AND "heat"[All Fields]) OR "extreme heat"[All Fields]) AND ("epidemiology"[MeSH Subheading] OR "epidemiology"[All Fields] OR "morbidity"[All Fields] OR "morbidity"[MeSH Terms] OR "morbid"[All Fields] OR "morbidities"[All Fields] OR "morbids"[All Fields]) AND ("emergency service, hospital"[MeSH Terms] OR ("emergency"[All Fields] AND "service"[All Fields] AND "hospital"[All Fields]) OR "hospital emergency service"[All Fields] OR ("emergency"[All Fields] AND "department"[All Fields]) OR "emergency department"[All Fields])) AND (2002:2022[pdat])  #9 (("extreme heat"[MeSH Terms] OR ("extreme"[All Fields] AND "heat"[All Fields]) OR "extreme heat"[All Fields]) AND ("epidemiology"[MeSH Subheading] OR "epidemiology"[All Fields] OR "morbidity"[All Fields] OR "morbidity"[MeSH Terms] OR "morbid"[All Fields] OR "morbidities"[All Fields] OR "morbids"[All Fields]) AND ("hospital s"[All Fields] OR "hospitalisation"[All Fields] OR "hospitalization"[MeSH Terms] OR "hospitalization"[All Fields] OR "hospitalising"[All Fields] OR "hospitality"[All Fields] OR "hospitalisations"[All Fields] OR "hospitalised"[All Fields] OR "hospitalizations"[All Fields] OR "hospitalized"[All Fields] OR "hospitalize"[All Fields] OR "hospitalizing"[All Fields] OR "hospitals"[MeSH Terms] OR "hospitals"[All Fields] OR "hospital"[All Fields])) AND (2002:2022[pdat])  #10 (("extreme heat"[MeSH Terms] OR ("extreme"[All Fields] AND "heat"[All Fields]) OR "extreme heat"[All Fields]) AND ("health services"[MeSH Terms] OR ("health"[All Fields] AND "services"[All Fields]) OR "health services"[All Fields])) AND (2002:2022[pdat])  **#11** (("extreme heat"[MeSH Terms] OR ("extreme"[All Fields] AND "heat"[All Fields]) OR "extreme heat"[All Fields]) AND ("outpatient s"[All Fields] OR "outpatients"[MeSH Terms] OR "outpatients"[All Fields] OR "outpatient"[All Fields]) AND ("visit"[All Fields] OR "visitation"[All Fields] OR "visitations"[All Fields] OR "visited"[All Fields] OR "visiting"[All Fields] OR "visits"[All Fields])) AND (2002:2022[pdat]) |
| --- | --- |
| **WOS/SCOPUS/PUBMED** |  |

**S3: 152 Developing country list according to IMF ^1^**

| [Afghanistan](https://www.worlddata.info/asia/afghanistan/index.php) | [Grenada](https://www.worlddata.info/america/grenada/index.php) | [Peru](https://www.worlddata.info/america/peru/index.php) |
| --- | --- | --- |
| [Albania](https://www.worlddata.info/europe/albania/index.php) | [Guatemala](https://www.worlddata.info/america/guatemala/index.php) | [Philippines](https://www.worlddata.info/asia/philippines/index.php) |
| [Algeria](https://www.worlddata.info/africa/algeria/index.php) | [Guinea](https://www.worlddata.info/africa/guinea/index.php) | [Poland](https://www.worlddata.info/europe/poland/index.php) |
| [Angola](https://www.worlddata.info/africa/angola/index.php) | [Guinea-Bissau](https://www.worlddata.info/africa/guinea-bissau/index.php) | [Qatar](https://www.worlddata.info/asia/qatar/index.php) |
| [Antigua and Barbuda](https://www.worlddata.info/america/antigua-barbuda/index.php) | [Guyana](https://www.worlddata.info/america/guyana/index.php) | [Republic of the Congo](https://www.worlddata.info/africa/congo-brazzaville/index.php) |
| [Argentina](https://www.worlddata.info/america/argentina/index.php) | [Haiti](https://www.worlddata.info/america/haiti/index.php) | [Romania](https://www.worlddata.info/europe/romania/index.php) |
| [Armenia](https://www.worlddata.info/asia/armenia/index.php) | [Honduras](https://www.worlddata.info/america/honduras/index.php) | [Russia](https://www.worlddata.info/europe/russia/index.php) |
| [Aruba](https://www.worlddata.info/america/aruba/index.php) * | [India](https://www.worlddata.info/asia/india/index.php) | [Rwanda](https://www.worlddata.info/africa/rwanda/index.php) |
| [Azerbaijan](https://www.worlddata.info/asia/azerbaijan/index.php) | [Indonesia](https://www.worlddata.info/asia/indonesia/index.php) | [Saint Kitts and Nevis](https://www.worlddata.info/america/stkitts-nevis/index.php) |
| [Bahamas](https://www.worlddata.info/america/bahamas/index.php) | [Iran](https://www.worlddata.info/asia/iran/index.php) | [Saint Lucia](https://www.worlddata.info/america/saint-lucia/index.php) |
| [Bahrain](https://www.worlddata.info/asia/bahrain/index.php) | [Iraq](https://www.worlddata.info/asia/iraq/index.php) | [Saint Vincent and the Grenadines](https://www.worlddata.info/america/stvincent-grenadines/index.php) |
| [Bangladesh](https://www.worlddata.info/asia/bangladesh/index.php) | [Ivory Coast](https://www.worlddata.info/africa/ivory-coast/index.php) | [Samoa](https://www.worlddata.info/oceania/samoa/index.php) |
| [Barbados](https://www.worlddata.info/america/barbados/index.php) | [Jamaica](https://www.worlddata.info/america/jamaica/index.php) | [Sao Tome and Principe](https://www.worlddata.info/africa/sao-tome-and-principe/index.php) |
| [Belarus](https://www.worlddata.info/europe/belarus/index.php) | [Jordan](https://www.worlddata.info/asia/jordan/index.php) | [Saudi Arabia](https://www.worlddata.info/asia/saudi-arabia/index.php) |
| [Belize](https://www.worlddata.info/america/belize/index.php) | [Kazakhstan](https://www.worlddata.info/asia/kazakhstan/index.php) | [Senegal](https://www.worlddata.info/africa/senegal/index.php) |
| [Benin](https://www.worlddata.info/africa/benin/index.php) | [Kenya](https://www.worlddata.info/africa/kenya/index.php) | [Serbia](https://www.worlddata.info/europe/serbia/index.php) |
| [Bhutan](https://www.worlddata.info/asia/bhutan/index.php) | [Kiribati](https://www.worlddata.info/oceania/kiribati/index.php) | [Seychelles](https://www.worlddata.info/africa/seychelles/index.php) |
| [Bolivia](https://www.worlddata.info/america/bolivia/index.php) | [Kosovo](https://www.worlddata.info/europe/kosovo/index.php) | [Sierra Leone](https://www.worlddata.info/africa/sierra-leone/index.php) |
| [Bosnia and Herzegovina](https://www.worlddata.info/europe/bosnia-and-herzegovina/index.php) | [Kuwait](https://www.worlddata.info/asia/kuwait/index.php) | [Solomon Islands](https://www.worlddata.info/oceania/solomon-islands/index.php) |
| [Botswana](https://www.worlddata.info/africa/botswana/index.php) | [Kyrgyzstan](https://www.worlddata.info/asia/kyrgyzstan/index.php) | [Somalia](https://www.worlddata.info/africa/somalia/index.php) |
| [Brazil](https://www.worlddata.info/america/brazil/index.php) | [Laos](https://www.worlddata.info/asia/laos/index.php) | [South Africa](https://www.worlddata.info/africa/south-africa/index.php) |
| [Brunei](https://www.worlddata.info/asia/brunei/index.php) | [Lebanon](https://www.worlddata.info/asia/lebanon/index.php) | [South Sudan](https://www.worlddata.info/africa/south-sudan/index.php) |
| [Bulgaria](https://www.worlddata.info/europe/bulgaria/index.php) | [Lesotho](https://www.worlddata.info/africa/lesotho/index.php) | [Sri Lanka](https://www.worlddata.info/asia/sri-lanka/index.php) |
| [Burkina Faso](https://www.worlddata.info/africa/burkina-faso/index.php) | [Liberia](https://www.worlddata.info/africa/liberia/index.php) | [Sudan](https://www.worlddata.info/africa/sudan/index.php) |
| [Burundi](https://www.worlddata.info/africa/burundi/index.php) | [Libya](https://www.worlddata.info/africa/libya/index.php) | [Suriname](https://www.worlddata.info/america/suriname/index.php) |
| [Cambodia](https://www.worlddata.info/asia/cambodia/index.php) | [Madagascar](https://www.worlddata.info/africa/madagascar/index.php) | [Syria](https://www.worlddata.info/asia/syria/index.php) |
| [Cameroon](https://www.worlddata.info/africa/cameroon/index.php) | [Malawi](https://www.worlddata.info/africa/malawi/index.php) | [Tajikistan](https://www.worlddata.info/asia/tajikistan/index.php) |
| [Cape Verde](https://www.worlddata.info/africa/cape-verde/index.php) | [Malaysia](https://www.worlddata.info/asia/malaysia/index.php) | [Tanzania](https://www.worlddata.info/africa/tanzania/index.php) |
| [Central African Republic](https://www.worlddata.info/africa/central-african-republic/index.php) | [Maldives](https://www.worlddata.info/asia/maldives/index.php) | [Thailand](https://www.worlddata.info/asia/thailand/index.php) |
| [Chad](https://www.worlddata.info/africa/chad/index.php) | [Mali](https://www.worlddata.info/africa/mali/index.php) | [Togo](https://www.worlddata.info/africa/togo/index.php) |
| [Chile](https://www.worlddata.info/america/chile/index.php) | [Marshall Islands](https://www.worlddata.info/oceania/marshall-islands/index.php) | [Tonga](https://www.worlddata.info/oceania/tonga/index.php) |
| [China](https://www.worlddata.info/asia/china/index.php) | [Mauritania](https://www.worlddata.info/africa/mauritania/index.php) | [Trinidad and Tobago](https://www.worlddata.info/america/trinidad-and-tobago/index.php) |
| [Colombia](https://www.worlddata.info/asia/china/index.php) | [Mauritius](https://www.worlddata.info/africa/mauritius/index.php) | [Tunisia](https://www.worlddata.info/africa/tunisia/index.php) |
| [Comoros](https://www.worlddata.info/africa/comoros/index.php) | [Mexico](https://www.worlddata.info/america/mexico/index.php) | [Turkey](https://www.worlddata.info/asia/turkey/index.php) |
| [Costa Rica](https://www.worlddata.info/america/costa-rica/index.php) | [Moldova](https://www.worlddata.info/europe/moldova/index.php) | [Turkmenistan](https://www.worlddata.info/asia/turkmenistan/index.php) |
| [Democratic Republic of the Congo](https://www.worlddata.info/africa/congo-kinshasa/index.php) | [Mongolia](https://www.worlddata.info/asia/mongolia/index.php) | [Tuvalu](https://www.worlddata.info/oceania/tuvalu/index.php) |
| [Djibouti](https://www.worlddata.info/africa/djibouti/index.php) | [Montenegro](https://www.worlddata.info/europe/montenegro/index.php) | [Uganda](https://www.worlddata.info/africa/uganda/index.php) |
| [Dominica](https://www.worlddata.info/america/dominica/index.php) | [Morocco](https://www.worlddata.info/africa/morocco/index.php) | [Ukraine](https://www.worlddata.info/europe/ukraine/index.php) |
| [Dominican Republic](https://www.worlddata.info/america/dominican-republic/index.php) | [Mozambique](https://www.worlddata.info/africa/mozambique/index.php) | [Uruguay](https://www.worlddata.info/america/uruguay/index.php) |
| [East Timor](https://www.worlddata.info/asia/east-timor/index.php) | [Myanmar](https://www.worlddata.info/asia/burma/index.php) | [Uzbekistan](https://www.worlddata.info/asia/uzbekistan/index.php) |
| [Ecuador](https://www.worlddata.info/america/ecuador/index.php) | [Namibia](https://www.worlddata.info/africa/namibia/index.php) | [Vanuatu](https://www.worlddata.info/oceania/vanuatu/index.php) |
| [Egypt](https://www.worlddata.info/africa/egypt/index.php) | [Nauru](https://www.worlddata.info/oceania/nauru/index.php) | [Venezuela](https://www.worlddata.info/america/venezuela/index.php) |
| [El Salvador](https://www.worlddata.info/america/el-salvador/index.php) | [Nepal](https://www.worlddata.info/asia/nepal/index.php) | [Vietnam](https://www.worlddata.info/asia/vietnam/index.php) |
| [Equatorial Guinea](https://www.worlddata.info/africa/equatorial-guinea/index.php) | [Nicaragua](https://www.worlddata.info/america/nicaragua/index.php) | [Yemen](https://www.worlddata.info/asia/yemen/index.php) |
| [Eritrea](https://www.worlddata.info/africa/eritrea/index.php) | [Niger](https://www.worlddata.info/africa/niger/index.php) | [Zambia](https://www.worlddata.info/africa/zambia/index.php) |
| [Eswatini](https://www.worlddata.info/africa/eswatini/index.php) | [Nigeria](https://www.worlddata.info/africa/nigeria/index.php) | [Zimbabwe](https://www.worlddata.info/africa/zimbabwe/index.php) |
| [Ethiopia](https://www.worlddata.info/africa/ethiopia/index.php) | [North Macedonia](https://www.worlddata.info/europe/northmacedonia/index.php) |  |
| [Federated States of Micronesia](https://www.worlddata.info/oceania/micronesia/index.php) | [Oman](https://www.worlddata.info/asia/oman/index.php) |  |
| [Fiji](https://www.worlddata.info/oceania/fiji/index.php) | [Pakistan](https://www.worlddata.info/asia/pakistan/index.php) |  |
| [Gabon](https://www.worlddata.info/africa/gabon/index.php) | [Palau](https://www.worlddata.info/oceania/palau/index.php) |  |
| [Gambia](https://www.worlddata.info/africa/gambia/index.php) | [Panama](https://www.worlddata.info/america/panama/index.php) |  |
| [Georgia](https://www.worlddata.info/asia/georgia/index.php) | [Papua New Guinea](https://www.worlddata.info/oceania/papua-new-guinea/index.php) |  |
| [Ghana](https://www.worlddata.info/africa/ghana/index.php) | [Paraguay](https://www.worlddata.info/america/paraguay/index.php) |  |

**S4: Checklist for the Risk of Bias Assessment according to The Navigation Guide ^2^**

| **Risk of bias**  **- will be assesses for each individual article ^3^** | |
| --- | --- |
| **Human study domains**   - Selection / recruitment strategy - Confounding - Exposure assessment - Outcome assessment - Selective outcome reporting - Conflict of interest - Other source of bias | **Determination for each of bias domain**   - Low risk - Probably low risk - Probably high risk - High risk |
| **Quality of evidence**  **- will be assessed using GRADE, in which the rating will be started as moderate quality, subsequently may be downgraded (-1 or -2) or upgraded (+1 or +2) according to factors ^4^** | |
| **Downgrade factors domain**   - Individual study limitations - Inconsistency of results - Indirectness of evidence - Imprecision - Publication bias | **Final rating (based on all quality factors)**   - High quality - Moderate quality - Low quality |
| **Upgrading factors domain**   - Dose-response gradient - Large magnitude of effect - Confounding |  |
| **Strength of evidence**  **- overall strength of evidence across all studies will be assessed based on the following criteria and the final rating implies to the level of certainty of toxicity ^5^** | |
| **Factors:**   - Quality of body of evidence - Direction of effect estimates - Confidence in effect estimates - Other attributes that possibly affect the certainty | **Final considerations level of certainty of toxicity**   - Sufficient - Limited - Inadequate - Lack of evidence |

**References**

1. IMF. *World Economic Outlook. A rocky recovery.* USA2023.

2. Woodruff TJ, Sutton P. The Navigation Guide Systematic Review Methodology: A Rigorous and Transparent Method for Translating Environmental Health Science into Better Health Outcomes. *Environmental Health Perspectives.* 2014;122(10):1007-1014.

3. National Toxicology Program. OHAT risk of bias tool. <https://ntp.niehs.nih.gov/ntp/ohat/pubs/riskofbiastool_508.pdf>. Published 2022. Accessed 16th november, 2022.

4. Balshem H, Helfand M, Schünemann HJ, et al. GRADE guidelines: 3. Rating the quality of evidence. *J Clin Epidemiol.* 2011;64(4):401-406.

5. Johnson PI, Sutton P, Atchley DS, et al. The Navigation Guide - evidence-based medicine meets environmental health: systematic review of human evidence for PFOA effects on fetal growth. *Environ Health Perspect.* 2014;122(10):1028-1039.
